# Examining the association between maternal prenatal psychiatric disorders and behavioural problems among offspring in early childhood: population-based study

**DOI:** 10.1192/bjo.2024.839

**Published:** 2025-01-17

**Authors:** Amy Braddon, Rosa Alati, Kim S. Betts

**Affiliations:** School of Population Health, Curtin University, Australia

**Keywords:** Electronic health records, neurodevelopmental disorders, big data, perinatal psychiatry, substance use disorders

## Abstract

**Background:**

Exposure to maternal mental illness during foetal development may lead to altered development, resulting in permanent changes in offspring functioning.

**Aims:**

To assess whether there is an association between prenatal maternal psychiatric disorders and offspring behavioural problems in early childhood, using linked health administrative data and the Australian Early Development Census from New South Wales, Australia.

**Method:**

The sample included all mother–child pairs of children who commenced full-time school in 2009 in New South Wales, and met the inclusion criteria (*N* = 69 165). Univariable logistic regression analysis assessed unadjusted associations between categories of maternal prenatal psychiatric disorders with indicators of offspring behavioural problems. Multivariable logistic regression adjusted the associations of interest for psychiatric categories and *a priori* selected covariates. Sensitivity analyses included adjusting the final model for primary psychiatric diagnoses and assessing association of interest for effect modification by child's biological gender.

**Results:**

Children exposed in the prenatal period to maternal psychiatric disorders had greater odds of being developmentally vulnerable in their first year of school. Children exposed to maternal anxiety disorders prenatally had the greatest odds for behavioural problems (adjusted odds ratio 1.98; 95% CI 1.43–2.69). A statistically significant interaction was found between child biological gender and prenatal hospital admissions for substance use disorders, for emotional subdomains, aggression and hyperactivity/inattention.

**Conclusions:**

Children exposed to prenatal maternal mental illness had greater odds for behavioural problems, independent of postnatal exposure. Those exposed to prenatal maternal anxiety were at greatest risk, highlighting the need for targeted interventions for, and support of, families with mental illness.

A growing body of research suggests that exposure to maternal stress during pregnancy has the potential to negatively influence development of the foetal central nervous system, leaving the individual at an increased susceptibility to a range of behavioural and developmental disorders in later life.^1,3^

## Prenatal influences on development

Elevated foetal cortisol concentration, resulting from maternal stress, may cause epigenetic dysregulation in offspring, resulting in persistent and abnormal hypothalamic-pituitary-adrenal axis functioning and serotonergic transmission in the offspring.^1,4^ Other studies have suggested that prenatal maternal stress alters the structure and function of the prefrontal cortex during the period of rapid brain development that occurs during pregnancy, with the high density of glucocorticoid receptors in the prefrontal cortex being key to this change.^4,7^ Importantly, the prefrontal cortex plays a role in the development of executive function, with alterations in executive function found to be associated with internalising and externalising disorders in childhood.^4^ Additionally, Donnici et al^8^ posits that alterations to the structure and function of the foetal amygdala, as a result of exposure to prenatal anxiety, may lead to behaviour problems later in childhood, considering the central role played by the amygdala in emotional regulation. All of these potential biological mechanisms are in some way linked to long-term outcomes regarding the emotional and behavioural development of the offspring through the life course.^4,7^

More recently, the focus has shifted from exposure to stressful life events in pregnancy to maternal mental health problems occurring therein, including depression, anxiety, substance use and psychosis. Studies have found exposure to maternal antenatal depression is associated with neurodevelopmental disorders such as autism and attention-deficit hyperactivity disorder.^9,11^ The effect on child development may vary based on the timing, severity, duration and type of illness the child is exposed to during foetal development.^12^ Within Australia, costs associated with maternal mental illness have been placed at $877 million annually.^13,14^ A recent national enquiry into mental health highlighted early identification and intervention for new parents as a priority reform.^14^ Reports on the prevalence of maternal perinatal mental illness, which focus predominately on the reporting of depression and anxiety, have varied across publications, ranging from 7 to 20%.^2,15,16^ Importantly, rates for prenatal mental health problems, as opposed to perinatal, have not been collected by population-based surveys, and thus numbers presented above are for the latter.

## Methodological issues

The nature and magnitude of associations between perinatal maternal mental disorders and child behavioural outcomes have varied across meta-analyses, with one meta-analysis^1^ finding maternal perinatal depression and anxiety predicted various developmental outcomes, including internalising and externalising behaviours, in offspring during the first 18 years. Conversely, a more recent meta-analysis^17^ found associations between exposure and language/cognitive development were neither clinically nor statistically significant. This meta-analysis also found a weak negative association between prenatal stress and/or anxiety and children's general intellectual development, and with associations varying based on the combination of exposures (i.e. maternal prenatal anxiety and/or stress).^17^ This implies that existing findings surrounding these association are inconsistent, thus necessitating further investigation. These meta-analyses suggest that an over-reliance on parent-reported measures for both maternal mental illness and measures of early childhood behavioural development, such as internalising and externalising behaviours, may partly explain the inconsistent findings.^4,12,17,18^ This conclusion was supported by a recent study that found associations between maternal mental health problems and childhood behavioural outcomes were stronger when based on parent-reported data compared with teacher-reported data.^12^ Considering these methodological issues, focusing on psychiatric hospital admissions could enhance the reliability of the findings through examining the more severe cases of prenatal mental illness, whereas the use of teacher-reported measures of child development may yield more consistent results.^4^

In this study, we utilised linked health administrative data to examine the association between maternal prenatal mental illness and offspring behavioural problems in early childhood. Prenatal maternal mental illness was measured by maternal psychiatric hospital admissions during the prenatal period, whereas offspring behavioural outcomes were obtained from the teacher-reported Australian Early Development Census (AEDC). Our findings further contribute to the understanding of the association between prenatal exposure to maternal mental illness and behavioural outcomes observed in offspring throughout early childhood.

## Method

### Sample

The sample includes all mother–offspring pairs (live births) born between 2003 and 2005 in the Australian state of New South Wales (NSW). The data for this study were obtained by linking individual records across the Perinatal Data Collection (PDC), Admitted Patient Data Collection (APDC) and AEDC, and covers all births within NSW, including public and private hospitals as well as home births. All live births and stillbirths of at least 20 weeks gestation or at least 400 g birth weight are included in the data (although the data used for this project excludes stillbirths). The 2009 AEDC was administered to 87 169 children in NSW, representing 99.9% of eligible children who commenced full-time school.^19^ Record linkage with maternal data was available for 74 863 mother–offspring pairs. Children were excluded from our analyses if they were identified within the AEDC as requiring additional assistance within the classroom for medical, physical or intellectual reasons (*n* = 3108); were missing scores from one or more AEDC domains (*n* = 516) and if they were born as part of a multiple birth (*n* = 2044). Children identified as having ‘special needs’ are those diagnosed with a physical or intellectual disability within their school records. As these children have already been identified as requiring additional assistance, AEDC category scores are not calculated.^20^ The final population sample available for analysis was 69 165 mother–offspring pairs. In NSW, health administrative linkage is undertaken by the Centre for Health Record Linkage (CHeReL), which is managed by the NSW Ministry of Health. All records within the datasets are de-identified in line with ethical considerations. The CHeReL maintains a Master Linkage Key that links patients across multiple data collections, including those used in this study, with the data-sets linked by using probabilistic linkage (Choicemaker software for Windows, Princeton, New Jersey, USA; https://www.choicemaker.com/Download).^21^ The data-sets used for this study are as follows.

### The outcome variables

The AEDC is a population-based census of children's development on entry to their first year of full-time school, which is conducted every 3 years. The results used for this study are from the 2009 AEDC. The AEDC is completed by teachers using the Australian version of the Early Development Instrument (AvEDI) for each child in their class.^22^ The AvEDI was adapted from the Early Development Instrument that was developed by McMaster University in Ottawa, Canada.^23^ The AEDC measures five domains, including physical health and well-being, social competence, emotional, language and cognitive skills (school-based), and communication skills and general knowledge.^22^ Teachers complete the AEDC for each student after having known the children for at least 1 month. The AEDC was completed nationally between 1 May and 31 July 2009, with information collected on 261 147 children across 7422 schools.^22^ Within the state of NSW, children are able to commence full-time schooling if they are turning 5 years old before July 31 of that year, with schooling becoming compulsory from 6 years of age.^24^

The emotional maturity domain was used as a measure for behavioural outcomes, and contains 26 items completed by the child's teacher, with all questions scored using a three-point scale: often or very true (10), sometimes or somewhat true (5), and never or not true (0).^23,25^ Within the emotional maturity domain, the scores are then collated within four behavioural subdomains, including pro-social and helping behaviour, anxious and fearful behaviour, aggressive behaviour and hyperactivity and inattention.^22^ Each of these four subdomain scores are further dichotomised into developmentally vulnerable indicator variables (yes/no) according to cut-offs, with children who score below the tenth percentile (in the lowest 10%) classified as ‘developmentally vulnerable’, and those scoring between the 11th and 25th percentiles being classified as developmentally at risk.^22^ These subdomains can be grouped into internalising problems, defined in the context of emotion regulation, such as anxious and fearful behaviour, and pro-social behaviours, whereas externalising problems can be defined as behaviours such as aggression and conduct disorders, including hyperactivity and inattention.^26,27^

### Exposure variables

The exposure variables were taken from the APDC, which contains diagnoses classified using the ICD-10, Australian Modification (ICD-10-AM), and procedures and interventions classified using the Australian Classification of Health Interventions. The data includes all in-patient separations, including transfers, discharges and deaths from all private hospitals, private day procedure centres, public hospitals, public psychiatric hospitals, and multi-purpose services in NSW, with roughly 400 facilities contributing to the data collection.^28^ Exposure was classified as a recorded admission with a primary or secondary diagnosis of an index psychiatric disorder occurring within the pregnancy period (i.e. after conception but before delivery – with conception date calculated as the delivery date minus the gestational age at delivery). The disorders include substance use disorders (SUD) (ICD-10 codes F10–F19), psychotic disorders (ICD-10 codes F20–F29), affective disorders (ICD-10 codes F30–F39), anxiety disorders (ICD-10 codes F40–F41).^29^ We combined affective (including bipolar disorder) (ICD-10 codes F30–F39) and psychotic disorders (ICD-10 codes F20–29) into a severe mental illness (SMI) category. The literature contains varied definitions of SMI, including measures of global assessment of functioning scores, encompassing ICD-10 codes F2x (schizophrenia, schizotypal, delusional and other non-mood psychotic disorders) and F3x (mood affective disorders).^30,31^ Lastly, mental health-related admissions were classified as prenatal exposure if they occurred in the period during pregnancy (i.e. after conception but before delivery – with conception date calculated as the delivery date minus the gestational age at delivery), with postnatal exposure being classified as any mental health-related admission occurring in the 12 months following the child's birth.

### Potential confounders

Potential confounding variables were taken from the PDC, and included maternal smoking status; birth weight; gestational age; Appearance, Pulse, Grimace, Activity and Respiration (APGAR) score for 1 and 5 mins; admission to neonatal intensive care unit or special care unit; and whether the child had to be resuscitated.^32^ The APGAR score is derived from assessment of a newborn's heart rate, respiratory effort, muscle tone, reflex irritability and colour and is a widely used measure to assess newborn health at birth.^33,34^

### Statistical analyses

Univariable logistic regression analysis was used to assess unadjusted associations (as odds ratio (95% confidence interval) between the predictors (i.e. categories of maternal psychiatric diagnoses) and the *a priori* confounders with each indicator of offspring behavioural problem (i.e. separate models for each dichotomous outcome). Next, multivariable logistic regression was used to adjust the associations for all the exposure variables. The *a priori* confounders were identified in previous research,^35,36^ and included maternal smoking status, maternal age, obstetric outcomes, maternal substance use, child age on school entry and child birth weight. Covariates identified as being statistically significantly associated with each indicator of offspring behavioural problems were included in the multivariable logistic regression.

Next, associations between all primary and secondary prenatal and postnatal psychiatric hospital admissions were assessed with the phi coefficient.

Lastly, a number of sensitivity analyses were conducted, including substituting primary/secondary maternal psychiatric diagnoses for primary diagnoses only, and testing for a gender-specific interaction affect in the association of interest.

All analyses were conducted with open-source software, R, version 4.2.2, for Windows 11 Home,^37^ with statistical significance set at <0.05.

### Ethics and consent statement

The project received ethical approval from NSW Population and Health Services Research Ethics Committee (approval number 2019/ETH01592) and Curtin University Human Research Office (approval number HRE2019-0601). The use of de-identified administrative data did not require informed consent from participants in NSW, as approved by the relevant ethics committees.

## Results

### Population

Children within the study had a mean age of 5.2 years (±0.35 years), and mothers had a mean age of 29.68 years (±5.54 years). First-time mothers accounted for 41.83% of the sample. English was spoken as a second language within the households of 15.22% of children. The prevalence and frequencies for primary prenatal diagnoses of anxiety disorder was 0.05% (*n* = 38), SUD was 0.03% (*n* = 22) and SMI was 0.09% (*n* = 60); the prevalence of primary postnatal diagnoses of anxiety disorder was 0.27% (*n* = 184), SUD was 0.09% (*n* = 61) and SMI was 0.32% (*n* = 218). Prevalence and frequencies for primary and secondary diagnoses are presented in Table 1.

**Table 1:** Prevalence and unadjusted odds ratios for covariates and maternal psychiatric diagnoses, for the sample of 69 165 mothers and their children

Covariates	Total population (*N* = 69 165)	Prenatal anxiety				Prenatal severe mental illness				Prenatal substance			
	% (*n*)	0.47 (325)				0.7 (485)				1.09 (756)			
		No, % (*n*)	Yes, % (*n*)	Odds ratio (95% CI)	*P*-value	No, % (*n*)	Yes, % (*n*)	Odds ratio (95% CI)	*P*-value	No, % (*n*)	Yes, % (*n*)	Odds ratio (95% CI)	*P*-value
Maternal age at child's birth <25 years	22.93 (15 862)	99.51 (15 785)	0.49 (77)	1.04 (0.8–1.34)	0.744	99.04 (15 710)	0.96 (152)	1.54 (1.27–1.86)	<0.001	97.88 (15 525)	2.12 (337)	2.74 (2.37–3.16)	<0.001
Maternal prenatal smoking	14.62 (10 115)	99.33 (10 047)	0.82 (83)	1.48 (1.26–1.69)	<0.001	98.18 (9931)	1.83 (184)	1.79 (1.61–1.98)	<0.001	93.62 (9470)	6.38 (645)	13.52 (11.57–15.84)	<0.001
Admitted to special care nursery	13.24 (9154)	99.26 (9086)	0.74 (68)	1.24 (1.05–1.4)	0.004	98.81 (9045)	1.20 (109)	1.27 (1.12–1.41)	<0.001	96.62 (8845)	3.38 (309)	1.53 (1.43–1.62)	<0.001
Admitted to NICU	1.77 (1226)	99.27 (1217)	0.73 (9)	1.27 (0.69–1.71)	0.270	99.02 (1214)	0.99 (12)	1.21 (0.71–1.62)	0.356	95.92 (1176)	4.08 (50)	1.73 (1.45–2.11)	<0.001
Preterm birth (<37 weeks gestation)	4.64 (3207)	98.97 (3174)	1.03 (33)	2.34 (1.6–3.3)	<0.001	98.38 (3155)	1.64 (52)	2.49 (1.85–3.3)	<0.001	95.32 (3057)	4.68 (150)	5.29 (4.4–6.33)	<0.001
Child's gender (male)	50.48 (34 915)	99.50 (34 742)	0.50 (173)	1	Reference	99.33 (34 682)	0.67 (233)	1	Reference	98.97 (34 554)	1.03 (361)	1	Reference
Child's gender (female)	49.52 (34 250)	99.56 (34 098)	0.46 (158)	0.9 (0.72–1.11)	0.320	99.26 (33 998)	0.74 (252)	1.1 (0.92–1.32)	0.281	98.85 (33 855)	1.15 (395)	1.12 (0.97–1.29)	0.131
Child ESL	15.22 (10 530)	99.66 (10 494)	0.34 (36)	0.69 (0.48–0.97)	0.037	99.79 (10 508)	0.21 (22)	0.26 (0.17–0.39)	<0.001	99.70 (10 498)	0.30 (32)	0.24 (0.17–0.34)	<0.001
Child's age <5 years	25.50 (17 637)	99.50 (17 548)	0.50 (89)	1.1 (0.86–1.4)	0.435	99.13 (17 483)	0.88 (154)	1.36 (1.12–1.65)	0.002	98.89 (17 441)	1.11 (196)	1.02 (0.87–1.2)	0.787

Child's gender (male) is the reference group for which child's gender (female) odds ratio is calculated. NICU, Neonatal Intensive Care Unit; ESL, English as second language.

### Descriptive statistics

Associations between maternal psychiatric hospital admissions, and covariates for the 69 165 child and mother pairs are presented in Table 1. Unadjusted odds ratios estimate the association between each category of maternal psychiatric admission and covariates. Pregnant mothers were 13.5 times more likely to have been admitted to hospital for an SUD in the prenatal period if they had reported smoking during pregnancy (odds ratio 13.52; 95% CI 11.57–15.87), whereas the odds for hospital admission for prenatal anxiety was 1.48 (95% CI 1.26–1.69) and for SMI was 1.79 (95% CI 1.61–1.98), respectively. Pregnant mothers from an ESL background were less likely to have been admitted to hospital for any of the psychiatric illnesses, with significant odds ratios ranging from 0.24 to 0.69. Table 2 shows the associations (phi coefficient) between all pre- and postnatal psychiatric admissions. The strongest relationship was between prenatal SUD with postnatal SUD (0.26; *P* < 0.001) and postnatal SMI (0.26; *P* < 0.001).

**Table 2:** Correlation matrix between all primary and secondary pre- and postnatal psychiatric hospital admissions, shown as phi coefficients and *P*-values

Diagnoses	Prenatal substance use disorder	Prenatal SMI	Prenatal anxiety disorder	Postnatal substance use disorder	Postnatal SMI	Postnatal anxiety disorder
All prenatal substance use diagnoses (F10–F19)	1.00					
All prenatal SMI diagnoses (F20–F39)	0.10 <0.001	1.00				
All prenatal anxiety diagnoses (F40–F48)	0.04 <0.001	0.09 <0.001	1.00			
All postnatal substance use diagnoses (F10–F19)	0.26 <0.001	0.07 <0.001	0.03 <0.001	1.00		
All postnatal SMI diagnoses (F20–F39)	0.06 <0.001	0.14 <0.001	0.06 <0.001	0.26 <0.001	1.00	
All postnatal anxiety diagnoses (F40–F48)	0.02 <0.001	0.04 <0.001	0.05 <0.001	0.11 <0.001	0.21 <0.001	1.00

All codes refer to ICD-10 codes. SMI, severe mental illness.

### Regression analyses

Table 3 presents the unadjusted odds ratios for the association between child behavioural outcomes and maternal psychiatric admissions, and the covariates. Each of the prenatal psychiatric illnesses were found to be significantly predictive of the behavioural outcomes. Children of mothers who were admitted to hospital for an SUD in the prenatal period were found to have the highest odds of being classified as developmentally vulnerable in the emotional maturity domain (odds ratio 2.71; 95% CI 2.24–3.27). Across the behavioural subdomains, children of mothers who were admitted to hospital for an SUD in the prenatal period were found to have the highest odds of aggressive behaviour (odds ratio 3; 95% CI 2.52–3.57) and hyperactivity/inattention (odds ratio 2.93; 95% CI 2.48–3.45).

**Table 3:** Unadjusted odds ratios for univariate models estimating associations between risk exposures and emotional domain and subdomain categories

Variables	Vulnerable, odds ratio (95% CI)	*P-*value	Pro-social, odds ratio (95% CI)	*P-*value	Anxious/fearful, odds ratio (95% CI)	*P-*value	Aggressive, odds ratio (95% CI)	*P-*value	Hyperactivity/inattention, odds ratio (95% CI)	*P-*value
All prenatal substance use diagnoses (F10–F19)	2.71 (2.24–3.27)	<0.001	1.74 (1.39–2.15)	<0.001	1.69 (1.39–2.04)	<0.001	3 (2.52–3.57)	<0.001	2.93 (2.48–3.45)	<0.001
All prenatal SMI diagnoses (F20–F39)	1.74 (1.31–2.27)	<0.001	1.57 (1.17–2.07)	0.002	2.11 (1.68–2.63)	<0.001	1.78 (1.37–2.27)	<0.001	2.16 (1.72–2.68)	<0.001
All prenatal anxiety diagnoses (F40–F48)	2.34 (1.71–3.13)	<0.001	1.76 (1.24–2.42)	0.001	1.58 (1.16–2.11)	0.002	1.78 (1.29–2.40)	<0.001	2.22 (1.69–2.89)	<0.001
Covariates										
All postnatal substance use diagnoses (F10–F19)	2.90 (2.05–4.02)	<0.001	1.93 (1.29–2.79)	0.001	1.75 (1.22–2.44)	0.001	3.19 (2.31–4.32)	<0.001	2.86 (2.10–3.85)	<0.001
All postnatal SMI diagnoses (F20–F39)	1.82 (1.32–2.44)	<0.001	1.45 (1.02–1.99)	0.029	1.60 (1.20–2.09)	0.001	1.94 (1.45–2.55)	<0.001	1.77 (1.35–2.30)	<0.001
All postnatal anxiety diagnoses (F40–F48)	1.40 (1.02–1.88)	0.028	1.23 (0.88–1.67)	0.203	1.27 (0.96–1.65)	0.077	1.70 (1.30–2.20)	<0.001	1.35 (1.03–1.75)	0.024
Maternal age at child's birth <25 years	1.70 (1.6–1.81)	<0.001	1.44 (1.35–1.54)	<0.001	1.24 (1.17–1.31)	<0.001	1.78 (1.68–1.88)	<0.001	1.73 (1.64–1.82)	<0.001
Maternal smoking exposure, prenatal	1.71 (1.6–1.83)	<0.001	1.30 (1.22–1.38)	<0.001	1.41 (1.34–1.5)	<0.001	1.71 (1.61–1.82)	<0.001	1.78 (1.68–1.89)	<0.001
Child's gender assigned at birth (male)	0.28 (0.26–0.30)	<0.001	0.34 (0.32–0.37)	<0.001	0.78 (0.75–0.82)	<0.001	0.33 (0.31–0.35)	<0.001	0.29 (0.28–0.31)	<0.001
Child ESL	1.10 (1.02–1.19)	0.017	1.69 (1.58–1.81)	0.000	0.88 (0.82–0.95)	0.001	0.84 (0.78–0.91)	<0.001	0.94 (0.88–1.01)	0.085
Child's age (continuous)	0.75 (0.69–0.81)	<0.001	0.66 (0.60–0.71)	0.000	0.91 (0.85–0.97)	0.007	0.98 (0.91–1.05)	0.556	0.68 (0.63–0.73)	<0.001
Admitted to special care nursery	1.17 (1.11–1.23)	<0.001	1.10 (1.04–1.16)	0.001	1.07 (1.01–1.12)	0.012	1.13 (1.07–1.19)	<0.001	1.13 (1.07–1.18)	<0.001
Admitted to NICU	1.39 (1.21–1.60)	<0.001	1.17 (1–1.36)	0.034	1.18 (1.03–1.34)	0.012	1.17 (1–1.34)	0.032	1.37 (1.21–1.56)	<0.001
Preterm birth (<37 weeks gestation)	1.45 (1.28–1.63)	<0.001	1.16 (1.02–1.32)	0.023	1.21 (1.08–1.34)	0.001	1.43 (1.28–1.59)	<0.001	1.49 (1.34–1.64)	<0.001
Birth weight	1 (1–1)	<0.001	1 (1–1)	0.053	1 (1–1)	0.019	1 (1–1)	0.019	1 (1–1)	<0.001

SMI, severe mental illness; ESL, English as second language; NICU, neonatal intensive care unit.

### Adjusted model

Table 4 presents the adjusted odds ratios for the association between child behavioural outcomes and maternal psychiatric admission, and for perinatal and demographic factors. After adjustment, children of mothers who were admitted to hospital in the prenatal period with an anxiety disorder had the greatest odds of being classed as developmentally vulnerable (odds ratio 1.98; 95% CI 1.43–2.69), followed by children of mothers who were admitted to hospital for an SUD in the prenatal period (odds ratio 1.52; 95% CI 1.17–1.95). Children of mothers who were admitted to hospital with an SMI during the prenatal period had non-significant odds of being classed as developmentally vulnerable (odds ratio 1.25; 95% CI 0.93–1.67). In the final model (Table 4), a statistically significant interaction was found between the child's gender and prenatal SUD, with the emotional maturity subdomains aggressive (*P* = 0.034) and hyperactivity/inattention (*P* < 0.001). Compared with male offspring, females had greater odds of being classed as developmentally vulnerable for the aggressive subdomain (female odds ratio 2.45, 95% CI 1.78–3.30; male odds ratio 1.65, 95% CI 1.28–2.11) and hyperactive/inattentive subdomain (female odds ratio 2.34, 95% CI 1.75–3.10; male odds ratio 1.51, 95% CI 1.18–1.92).

**Table 4:** Adjusted odds ratios for full models estimating associations between all exposures and emotional maturity domain and subdomains

Variables	Vulnerable, odds ratio (95% CI)	*P-*value	Pro-social, odds ratio (95% CI)	*P-*value	Anxious/fearful, odds ratio (95% CI)	*P-*value	Aggressive, odds ratio (95% CI)	*P-*value	Hyperactivity/inattention, odds ratio (95% CI)	*P-*value
All prenatal substance use diagnoses (F10–F19)	1.52 (1.17–1.95)	0.001	1.49 (1.12–1.96)	0.006	1.23 (0.93–1.61)	0.141	1.64 (1.28–2.09)^a^	<0.001	1.44 (1.13–1.82)^a^	0.003
All prenatal SMI diagnoses (F20–F39)	1.25 (0.93–1.67)	0.132	1.38 (1.01–1.84)	0.037	1.77 (1.40–2.22)	<0.001	1.26 (0.95–1.65)	0.093	1.58 (1.24–2.01)	<0.001
All prenatal anxiety diagnoses (F40–F48)	1.98 (1.43–2.69)	<0.001	1.59 (1.11–2.21)	0.009	1.35 (0.99–1.81)	0.053	1.44 (1.03–1.97)	0.028	1.84 (1.38–2.43)	<0.001
Covariates										
Gender (male)	0.26 (0.24–0.28)	<0.001	0.33 (0.31–0.35)	<0.001	0.77 (0.74–0.81)	<0.001	0.32 (0.30–0.34)	<0.001	0.27 (0.26–0.29)	<0.001
Child's age (continuous)	0.67 (0.62–0.73)	<0.001	0.65 (0.60–0.71)	<0.001	0.87 (0.82–0.94)	0.001	0.86 (0.80–0.93)	<0.001	0.58 (0.54–0.63)	<0.001
Maternal age at child's birth <25 years	1.45 (1.45–1.64)	<0.001	1.34 (1.26–1.43)	<0.001	1.17 (1.10–1.23)	<0.001	1.64 (1.55–1.74)	<0.001	1.57 (1.48–1.65)	<0.001
Maternal smoking exposure, prenatal	1.51 (1.42–1.62)	<0.001	1.26 (1.18–1.35)	<0.001	1.33 (1.25–1.40)	<0.001	1.45 (1.37–1.55)	<0.001	1.52 (1.43–1.61)	<0.001
Admitted to special care nursey	1.09 (1.03–1.16)	0.004	1.05 (0.98–1.11)	0.163	1.03 (0.98–1.09)	0.245	1.07 (1.01–1.14)	0.019	1.05 (0.99–1.10)	0.121
Admitted to NICU	1.21 (1.04–1.40)	0.012	1.06 (0.88–1.24)	0.535	1.12 (0.97–1.28)	0.115	1.02 (0.86–1.19)	0.849	1.21 (1.06–1.38)	0.005
Birth weight	1 (1–1)	<0.001	1 (1–1)	0.009	1 (1–1)	0.279	1 (1–1)	0.032	1 (1–1)	<0.001
Child ESL	1.07 (0.98–1.16)	0.123	1.60 (1.49–1.73)	<0.001	0.89 (0.83–0.96)	0.002	0.84 (0.78–0.92)	<0.001	0.89 (0.82–0.95)	0.001
All postnatal substance use diagnoses (F10–F19)	1.34 (0.90–1.95)	0.142	1.26 (0.80–1.92)	0.305	1.06 (0.72–1.54)	0.747	1.35 (0.94–1.92)	0.101	1.26 (0.89–1.78)	0.187
All postnatal SMI diagnoses (F20–F39)	1.23 (0.85–1.72)	0.248	1.16 (0.79–1.66)	0.426	1.20 (0.88–1.62)	0.239	1.26 (0.90–1.72)	0.163	1.16 (0.85–1.57)	0.331
All postnatal anxiety diagnoses (F40–F48)	1.20 (0.86–1.64)	0.278	1.13 (0.80–1.56)	0.468	1.14 (0.86–1.50)	0.353	1.50 (1.12–1.97)	0.004	1.17 (0.88–1.55)	0.267

SMI, severe mental illness; NICU, neonatal intensive care unit; ESL, English as second language.

a. A statistically significant interaction was found between child gender assigned at birth and prenatal hospital admissions for substance use disorders, for emotional subdomains aggressive (odds ratio 1.48, 95% CI 1.03–2.12) and hyperactivity/inattention (odds ratio 1.78, 95% CI 1.26–2.50).

Table 5 presents results from the sensitivity analysis, using primary diagnoses After restricting hospital psychiatric admissions to primary diagnoses only, the increased odds for developmental vulnerability based on prenatal psychiatric hospital admissions were no longer statistically significant. Conversely, postnatal hospital admissions for SMI were significantly associated with an increased odds for developmental vulnerability in the primary diagnoses model (odds ratio 2.14; 95% CI 1.43–3.10).

**Table 5:** Sensitivity analysis using hospital admissions with primary psychiatric diagnoses

Variables	Vulnerable, odds ratio (95% CI)	*P-*value	Pro-social, odds ratio (95% CI)	*P-*value	Anxious/fearful, odds ratio (95% CI)	*P-*value	Aggressive, odds ratio (95% CI)	*P-*value	Hyperactivity/inattention, odds ratio (95% CI)	*P-*value
All prenatal substance use primary diagnoses (F10–F19)	2.34 (0.49–8.72)	0.230	4.85 (1.21–17.47)	0.017	2.44 (0.51–9.01)	0.205	2.83 (0.69–10.39)	0.120	2.41 (0.60–8.69)	0.183
All prenatal SMI primary diagnoses (F20–F39)	1.66 (0.76–3.29)	0.173	1.47 (0.62–3.06)	0.333	1.50 (0.74–2.78)	0.228	1.09 (0.48–2.21)	0.832	1.50 (0.75–2.83)	0.230
All prenatal anxiety primary diagnoses (F40–F48)	1.37 (0.46–3.32)	0.523	1.35 (0.40–3.49)	0.578	1.60 (0.64–3.45)	0.266	1.42 (0.52–3.25)	0.443	1.94 (0.84–4.09)	0.098
Covariates										
Gender (male)	0.26 (0.25–0.28)	<0.001	0.33 (0.31–0.35)	<0.001	0.77 (0.73–0.81)	<0.001	0.32 (0.30–0.34)	<0.001	0.28 (0.26–0.29)	<0.001
Child's age (continuous)	0.67 (0.61–0.73)	<0.001	0.65 (0.60–0.71)	<0.001	0.87 (0.81–0.94)	<0.001	0.86 (0.79–0.93)	<0.001	0.58 (0.54–0.62)	<0.001
Maternal age at child's birth <25 years	1.55 (1.46–1.65)	<0.001	1.34 (1.26–1.43)	<0.001	1.17 (1.11–1.24)	<0.001	1.65 (1.56–1.75)	<0.001	1.57 (1.49–1.66)	<0.001
Maternal smoking exposure, prenatal	1.56 (1.46–1.67)	<0.001	1.29 (1.20–1.38)	<0.001	1.34 (1.27–1.42)	<0.001	1.51 (1.42–1.60)	<0.001	1.58 (1.49–1.68)	<0.001
Admitted to special care nursey	1.10 (1.04–1.17)	0.001	1.05 (0.99–1.12)	0.108	1.04 (0.98–1.09)	0.184	1.09 (1.02–1.15)	0.006	1.06 (1.00–1.12)	0.044
Admitted to NICU	1.22 (1.04–1.40)	0.009	1.06 (0.88–1.25)	0.502	1.12 (0.97–1.28)	0.114	1.03 (0.87–1.20)	0.741	1.22 (1.06–1.39)	0.004
Birth weight	1 (1–1)	<0.001	1 (1–1)	0.003	1 (1–1)	0.186	1 (1–1)	0.006	1 (1–1)	<0.001
Child ESL	1.06 (0.97–1.15)	0.174	1.59 (1.48–1.72)	<0.001	0.89 (0.83–0.96)	0.001	0.84 (0.77–0.91)	<0.001	0.88 (0.82–0.94)	<0.001
All postnatal substance use primary diagnoses (F10–F19)	0.93 (0.39–1.93)	0.853	0.87 (0.32–1.96)	0.754	0.68 (0.28–1.42)	0.343	2.10 (1.11–3.78)	0.017	1.18 (0.59–2.20)	0.630
All postnatal SMI primary diagnoses (F20–F39)	2.14 (1.43–3.10)	<0.001	1.49 (0.94–2.28)	0.076	1.77 (1.23–2.47)	0.001	2.12 (1.46–3.01)	<0.001	2.00 (1.40–2.81)	<0.001
All postnatal anxiety primary diagnoses (F40–F48)	0.84 (0.46–1.43)	0.550	0.88 (0.47–1.51)	0.670	1.06 (0.66–1.62)	0.784	1.02 (0.60–1.61)	0.974	0.74 (0.43–1.20)	0.246

SMI, severe mental illness; NICU, neonatal intensive care unit; ESL, English as second language.

## Discussion

This study utilised linked health administrative data to test the association between maternal prenatal mental illness and offspring behavioural problems in early childhood, adjusting for maternal postnatal mental illness and key confounders, in addition to assessing for effect modification by gender. We found that children of mothers who were admitted to hospital for prenatal anxiety were at the greatest increased odds for behavioural problems, followed by children of mothers who were admitted to hospital for prenatal SUD, whereas associations for prenatal SMI and emotion domain vulnerability were non-significant. Regarding gender-specific effects, we found a statistically significant interaction such that female children exposed to prenatal SUD experienced greater odds for externalising behavioural problems, compared with exposed male children. In summary, our findings support the hypothesis that prenatal exposure to maternal mental illness, independent of postnatal exposure, is associated with behavioural problems in early childhood, indicating an *in utero* mechanism.

Our findings corroborate those of both a current meta-analysis and a recent study,^1,38^ whereby an association between prenatal anxiety and hyperactivity/inattention behavioural problems were found. Importantly, these studies relied on self-reported and parent-reported measures, which are known to be biased, as mothers with elevated symptoms of anxiety may inaccurately report their child's behaviour.^39^ Therefore our results further contribute to the literature through the use of teacher-reported measures and hospital admission data, providing stronger evidence for this association.^38^ Critically, our results further support findings from a recent meta-analysis, whereby prenatal anxiety was associated with behavioural problems, independent of postnatal exposure, further supporting our hypothesis of an *in utero* effect.^1^

Our study utilised the same NSW population sample as two previous studies,^35,40^ but there were important methodological differences. Green et al^35^ investigated the link between maternal mental illness diagnosed before childbirth and AEDC domains, whereas our study focused specifically on prenatal diagnoses of mental illness and the association with vulnerability across the emotional development subdomains of the AEDC. Our adjusted model revealed that the association between prenatal anxiety and vulnerability in the emotional domain mirrored Green et al's findings regarding broad diagnoses of maternal illness pre-childbirth. However, we found that the association between prenatal mental illness and emotional vulnerability varied by diagnosis, with prenatal anxiety showing the highest odds (odds ratio 1.98; 95% CI 1.43–2.69), whereas associations with SMI were non-significant (odds ratio 1.25; 95% CI 0.93–1.67).

Additionally, Dean et al^40^ examined the influence of parental mental illness on internalising and externalising vulnerabilities in children, using the same AEDC subdomains (excluding the pro-social subdomain). Unlike Dean et al, who assessed diagnoses for both parents from 2000 to 2009, our study focused solely on maternal diagnoses during the prenatal and postnatal periods. Dean et al also identified a similar interaction between child gender assigned at birth and parental hospital admissions for SUDs, noting a stronger effect on hyperactive/inattentive vulnerabilities in female offspring compared with males. Building on the work by Green et al^35^ and Dean et al,^40^ our findings further explore this data-set, with a focus on prenatal diagnoses, and provide additional insight into the potential impact of prenatal maternal mental illness on child behavioural and emotional development.

It is important to highlight the inherent complexity of testing for an association with prenatal substance use exposure. Drugs have the capacity to cross and potentially alter the placental barrier, thereby directly affecting the developing foetus and exerting an influence on infant development.^41^ Although we observed a significant association between exposure to prenatal SUDs and various behavioural outcomes, it is important to recognise that prenatal substance use often co-occurs alongside other known risk factors for the outcome, including maternal childhood trauma, partner substance use and domestic violence,^41,42^ none of which we were able to control for.

Our analysis revealed an interaction between maternal prenatal SUDs and child gender, with exposed female children showing greater odds of externalising behavioural problems compared with males. Because of insufficient statistical power, we could not evaluate associations by specific substance exposures. Similar findings were reported in studies of prenatal cannabis^43^ and cocaine exposure.^44^ Finger et al^44^ suggested a biological mechanism involving autonomic regulation, specifically respiratory sinus arrhythmia, as a potential mediator between prenatal cocaine exposure and behavioural issues, indicating that female offspring may be more sensitive to cocaine's negative effects on self-regulation *in utero*. However Finger et al^44^ also acknowledged that these effects may be caused by other contextual or environmental factors, such as exposed children having experienced greater environmental adversity, which may contribute to the observed differences. Notably, the literature indicates varying gender effects depending on substance type, and as such, our results should be interpreted with caution.^45^

On the other hand, our findings were not consistent with a number of studies that found that the association of interest attenuated after adjusting for important confounders. Further, previous studies^46,47^ have demonstrated that although there are significant associations between both prenatal anxiety and prenatal depression with behavioural problems, increased duration of exposure across the prenatal and early childhood periods exerts a more pronounced effect on the outcome, suggesting that both prenatal and postnatal phases are critical for child development.

We found that the associations between prenatal SMI and the outcome were non-significant, which may be attributed to the heterogenous nature of the mental illnesses included in the SMI grouping. The low prevalence of these disorders within our data precluded us from testing for an association by discrete categories (e.g. psychotic disorders). Interestingly, in our sensitivity analysis, postnatal primary diagnosis of SMI was significantly associated with an increased odds of behavioural problems. Thus, the null findings for prenatal SMI may partially be attributed to the lack of statistical power when assessing prenatal SMI diagnoses.

Although there are few other population-based studies examining the association between prenatal maternal mental illness and behavioural problems in children, our results are consistent with Bell et al,^16^ who also used a population-based cohort. However the study by Bell et al did not differentiate between diagnoses of maternal prenatal mental illness (i.e. used any mental health diagnosis). In contrast to Bell et al, our study included both primary and secondary diagnoses in the exposure, enhancing our statistical power but potentially attenuating the results through the inclusion of hospital admissions not primarily related to maternal mental health. Notably, Bell et al's findings endorse the inclusion of secondary diagnoses by revealing a substantial increase in the odds of developmental vulnerability upon their incorporation.^16^

Our study further advances the literature by demonstrating the differing associations with child behavioural problems, based on maternal prenatal mental illness diagnoses. Strengths of our study include the use of linked health administrative data and teacher-reported child behavioural measures, whereby reducing the risk of reporting and recall bias. Further, it has been demonstrated that the subset of individuals with maternal records in the sample is reflective of the entire state of NSW, thus making it generally characteristic of the Australian population within this demographic.^19^

### Limitations

Despite the large sample used in our analysis, the rare exposure (in-patient psychiatric diagnoses) meant that we may have been underpowered to detect weak and moderate associations. This limitation underscores the need for using larger sample sizes and whole-population samples as we have done, to detect more subtle associations when using strict definitions of exposure and outcome. As a final point, multiple hypotheses were tested, which raises concerns around the risks of multiple comparison. However as this study was only exploratory, we did not correct for multiple testing.^48^

This study was unable to account for parental reports on child behaviour, which can provide a more comprehensive view of the child's behaviour across different contexts. This may have led to our results being downward biased, as a recent meta-analysis found associations between maternal mental health problems and childhood behavioural outcomes were larger when based on parent-reported data compared with teacher-reported data.^12^

We were unable to account for residual confounding, such as marital status, socioeconomic status, significant life events, physical and mental health comorbidities, family history and paternal data, all of which have been recognised within the literature as important contributors to early child development.^35,36^ Additionally, we were unable to control for familial confounders, with a study by Bekkhus et al finding that associations between prenatal anxiety exposure and behavioural problems in offspring were no longer significant after controlling for social and genetic confounders in the sibling comparison.^49^ Similarly, Gjerde et al found that after controlling for familial confounders, only concurrent depression was associated with behavioural problems.^50^ Further, our study could not account for the potential impact of maternal treatment (biological and/or psychological treatments) on both maternal mental health and child outcomes, leading to heterogeneity within our predictor. Importantly, research has suggested that psychological interventions for prenatal depression may be associated with improved stress reactivity in infants.^51^ Future studies are needed with the capacity to account for treatment type as a potential confounder for the association.

It is important to recognise that our data-set does not contain information regarding maternal psychiatric diagnoses from ambulatory services or primary care, only including those from in-patient care, which inherently includes the most severe cases. Consequently, this could result in underestimating the prevalence and impact of less severe, but still clinically significant psychiatric conditions, with the comparison group including children who have been exposed to maternal mental illness that did not require hospital admission, potentially downward biasing our results and reducing the generalisability of our findings.

### Significance

This study further contributes to the current literature examining the relationship between prenatal exposures and offspring development, by showing an association between prenatal maternal psychiatric disorders and offspring behavioural problems, using strict definitions of exposure and outcome. Notably, this research highlights the importance of early identification of mental health issues in people of childbearing age, as timely identification and intervention may lead to improved maternal and child outcomes. Clinically, this research underscores the need for healthcare providers to implement routine screenings for mental health, not only in prenatal care, but in people of childbearing age, ensuring those who are at risk receive appropriate support. Importantly, this study highlights that prenatal exposure to maternal mental health problems can have significant practical implications for child neurodevelopmental outcomes, underscoring the importance of early screening and support for both the mother and child. Future research within this area should look to explore the impact of familial, social and genetic confounders.

## Data Availability

The author has access to the data used for the study under a strict agreement with the NSW Ministry of Health and is not authorised to share the data. Accessing the data would require a genuine research organisation to enter into agreement with the NSW Ministry of Health and payment of fees.
